# Postorgasmic Side Effects of Tadalafil: A Case Report

**DOI:** 10.1002/ccr3.71033

**Published:** 2025-09-24

**Authors:** S. M. Yasir Arafat, Sadeed Hossain, Md Rokon Uddin

**Affiliations:** ^1^ Department of Psychiatry Bangladesh Specialized Hospital Dhaka Bangladesh; ^2^ Department of Public and Community Health, Faculty of Medicine and Health Sciences Frontier University Garowe Garowe Somalia; ^3^ Biomedical Research Foundation Dhaka Bangladesh; ^4^ Department of Dermatology and Laser Unit Bangladesh Specialized Hospital Dhaka Bangladesh

**Keywords:** Bangladesh, case report, PDE5 inhibitor, postorgasmic illness syndrome, tadalafil

## Abstract

We present a case of postorgasmic side effects related to the use of tadalafil 2.5 mg tablet. The patient developed symptoms like dizziness, palpitations, headache, and anxiety within minutes of ejaculation whenever he used tadalafil. Discontinuation of the drug led to complete resolution of all symptoms.

## 1 Introduction

Postorgasmic illness syndrome (POIS) is a rare, mysterious clinical condition characterized by flu‐like symptoms, muscle pain (myalgia), joint pain (arthralgia), muscle weakness, headache, dizziness, and depression or anxiety. These symptoms usually develop shortly after ejaculation and can persist up to 7 days [1, 2]. In 2002, Waldinger and Schweitzer first described the symptoms of a postejaculatory syndrome in two men with spontaneous ejaculations [3]. The pathophysiology of POIS still remains uncertain. There are several hypotheses on the pathogenesis of POIS, but a widely accepted hypothesis is proposed by Waldinger et al., in which POIS is considered an immunologic phenomenon as 88% of subjects had a positive Skin Prick Test reaction to their own semen, whereas none showed a skin reaction to the placebo [1, 4]. According to Waldinger, there are two types of POIS: one is the primary type, in which POIS manifests from the first ejaculation in puberty or adolescence; the other is the secondary type, in which POIS starts later in life [2]. Various research has been conducted to find out the causative factors and triggers of POIS, but no pharmacological causative factors or triggers have been identified yet [1].

Tadalafil is a phosphodiesterase‐5 (PDE5) inhibitor that is commonly used to treat erectile dysfunction [5]. Tadalafil is primarily indicated for the treatment of erectile dysfunction (ED), benign prostatic hyperplasia (BPH), and pulmonary arterial hypertension (PAH) [6, 7, 8]. Like many pharmacological agents, tadalafil has adverse effects. Some common adverse effects are headache, dyspepsia, myalgia, flushing, and nasal congestion. In rare cases, serious adverse reactions like sudden vision loss may occur where immediate medical attention is needed [9]. The incidence of adverse effects of tadalafil may occur differently in patient populations. Men who have radical prostatectomy (post‐RP) or radiation therapy (post‐RT) tend to have higher rates of adverse events compared to the general population suffering from erectile dysfunction [9]. Here, we report a case of postorgasmic side effects related to the use of tadalafil 2.5 mg tablet in Bangladesh mimicking POIS.

## 2 Case History/Examination

A 26‐year‐old married Bangladeshi man came to a private medical consultation chamber with recurrent episodes of marked fatigue, dizziness, headache, cognitive dullness, anxiety, and fear of impending death occurring within 30 min following ejaculation for the last 2 weeks. These symptoms persisted for approximately 1–2 h and resolved spontaneously without any medical intervention.

The patient is a garment worker and regular “gul” (smokeless tobacco) user. He uses gul after every intercourse. The patient reported no prior history of such symptoms in the 5 years of his marriage. He had been experiencing reduced penile rigidity during long‐time intercourse (30–40 min) since 2020 and had been intermittently using herbal medications that were able to relieve his condition. But the patient was seeking a permanent solution, so he consulted with a dermatologist who prescribed him tadalafil 2.5 mg, escitalopram 5 mg, and some vitamins.

The next day, he took tadalafil, followed by sexual intercourse. Within 15 min of ejaculation, he developed dizziness, palpitations, headache, and anxiety, all of which resolved spontaneously without any medical intervention within 2 h. The same symptoms recurred the following night, during which he also experienced a brief syncopal attack and fell down. He subsequently discontinued tadalafil for 4 days without stopping escitalopram 5 mg and vitamin, and during this period he did not have any symptoms. Then, on the 5th day he took half of tadalafil. This time postejaculatory symptoms recurred but were less severe and resolved within 10 min. Subsequently, he was taking tadalafil 2.5 mg tablet daily. He was afraid of the situation and avoiding ejaculation while performing intercourse for the fear of symptoms that prevented recurrence of symptoms. The symptoms reappeared for approximately 1 h after an unintentional ejaculation. Subsequently, he consulted with another dermatologist and a psychiatrist for the symptoms. He was suggested to stop tadalafil, and tablet sertraline 50 mg in the morning was prescribed instead of escitalopram 5 mg along with proper psychoeducation.

## 3 Methods (Differential Diagnosis, Investigations and Treatment)

He denied any past psychiatric illness, allergies, or chronic comorbidities. No abnormalities were found after physical examination. Laboratory investigations, including complete blood count, erythrocyte sedimentation rate, random blood glucose, serum creatinine, testosterone, and urine routine microscopy, were within normal limits. No signs of infection or other systemic conditions were found. Based on the symptom pattern, panic disorder could be considered as a differential diagnosis as the patient developed sudden symptoms, fear of impending death, fear of further episodes, and automatic resolution of the episodes. However, the history clearly indicates that the symptoms were related to ejaculation after taking tadalafil 2.5 mg tablet and resolved once the drug was stopped, which excludes the diagnosis.

## 4 Conclusions and Results (Outcome and Follow‐Up)

Tadalafil was stopped, and after discontinuation, the patient resumed intercourse without tadalafil and reported no recurrence of symptoms after a two‐week follow‐up.

## 5 Discussion

POIS remains a rare and poorly understood clinical condition, characterized by various physical and psychological symptoms that develop following ejaculation. Since its first description by Waldinger and Schweitzer [3], the pathophysiology of POIS is still unclear. Multiple theories have been proposed to explain its pathophysiology, but the most widely supported hypothesis involves an autoimmune or allergic response to seminal plasma components, as evidenced by positive skin prick tests with autologous semen in the majority of tested patients [2, 4]. However, a definitive causative factor has yet to be established.

This case is unique because the correlation between the initiation of tadalafil and the onset of POIS mimicking symptoms suggests a potential pharmacological trigger, which is something not previously reported in any literature. In the typical presentation of POIS, symptoms begin following ejaculation by intercourse or masturbation in adolescence or early adulthood without any clear precipitant [2], however, in this case, the patient had no prior history of such symptoms across 5 years of regular sexual activity. The sudden onset of POIS mimicking symptoms following tadalafil 2.5 mg tablet use and its complete resolution upon discontinuation of the drug presents a novel potential etiological link.

Tadalafil itself is not known to directly trigger immunological reactions to semen, but its pharmacological action may indirectly influence neurovascular or autonomic pathways that modulate both ejaculatory and immune responses. A phosphodiesterase type 5 (PDE5) inhibitor like tadalafil and sildenafil causes smooth muscle relaxation and vasodilation via the nitric oxide (NO)–cGMP pathway that can alter ejaculatory dynamics, which are under neurovascular and autonomic control, and affecting immune microenvironments by changing local or systemic blood flow [6, 10].

The patient also experienced a brief syncopal episode, which is rare in POIS and may represent a vasovagal response to autonomic dysregulation by the pharmacologic effects of tadalafil [11]. Importantly, his symptoms resolved completely after tadalafil withdrawal, and no recurrence was observed during drug‐free sexual activity, further strengthening the case for tadalafil‐associated postorgasmic side effects.

Since the incidence of POIS is very low and it is highly under‐reported, this case study serves as a reminder for clinicians to ask about recent medication use, particularly sexual performance enhancers, during the evaluation of patients with postejaculatory symptoms.

## 6 Conclusion

This case presentation shows tadalafil 2.5 mg tablet‐related postorgasmic side effects mimicking POIS. It suggests that tadalafil may act as a trigger in susceptible individuals, so clinicians should be aware of this potential risk of developing POIS after using PDE5 inhibitors like tadalafil. Further studies are necessary to find out the actual mechanisms of tadalafil that are responsible for these symptoms or to establish whether specific pharmacologic agents may trigger POIS‐mimicking symptoms.

## Author Contributions

**S. M. Yasir Arafat:** conceptualization, data curation, writing – original draft, writing – review and editing. **Sadeed Hossain:** writing – original draft, writing – review and editing. **Md Rokon Uddin:** data curation, writing – review and editing.

## Consent

Written informed consent was obtained from the patient for publication of this case report.

## Conflicts of Interest

The authors declare no conflicts of interest.

## Data Availability

The data that support the findings of this study are available on request from the corresponding author. The data are not publicly available due to privacy or ethical restrictions.

## References

[1] H. M. T. Nguyen , A. Bala , A. T. Gabrielson , and W. J. G. Hellstrom , “Post‐Orgasmic Illness Syndrome: A Review,” Sexual Medicine Reviews 6, no. 1 (2018): 11–15, 10.1016/j.sxmr.2017.08.006. 29128269

[2] M. D. Waldinger , “Post Orgasmic Illness Syndrome (POIS),” Translational Andrology and Urology 5, no. 4 (2016): 602–606, 10.21037/tau.2016.07.01. 27652231 PMC5001999

[3] M. D. Waldinger and D. H. Schweitzer , “Postorgasmic Illness Syndrome: Two Cases,” Journal of Sex & Marital Therapy 28, no. 3 (2002): 251–255, 10.1080/009262302760328280. 11995603

[4] M. D. Waldinger , M. M. H. M. Meinardi , A. H. Zwinderman , and D. H. Schweitzer , “Postorgasmic Illness Syndrome (POIS) in 45 Dutch Caucasian Males: Clinical Characteristics and Evidence for an Immunogenic Pathogenesis (Part 1),” Journal of Sexual Medicine 8, no. 4 (2011): 1164–1170, 10.1111/j.1743-6109.2010.02166.x. 21241453

[5] N. Pyrgidis , I. Mykoniatis , A. B. Haidich , et al., “The Effect of Phosphodiesterase‐Type 5 Inhibitors on Erectile Function: An Overview of Systematic Reviews,” Frontiers in Pharmacology 12 (2021): 735708, 10.3389/fphar.2021.735708. 34557099 PMC8452927

[6] R. M. Coward and C. C. Carson , “Tadalafil in the Treatment of Erectile Dysfunction,” Therapeutics and Clinical Risk Management 4, no. 6 (2008): 1315–1330, 10.2147/TCRM.S3336. 19337438 PMC2643112

[7] M. P. Curran , “Tadalafil: In the Treatment of Signs and Symptoms of Benign Prostatic Hyperplasia With or Without Erectile Dysfunction,” Drugs & Aging 29, no. 9 (2012): 771–781, 10.1007/s40266-012-0010-7. 23018613

[8] J. R. Klinger , “Tadalafil for the Treatment of Pulmonary Arterial Hypertension,” Expert Review of Respiratory Medicine 5, no. 3 (2011): 315–328, 10.1586/ers.11.38. 21702653

[9] A. L. Burnett , A. Nehra , R. H. Breau , et al., “Erectile Dysfunction: AUA Guideline,” Journal of Urology 200 (2018): 633–641. 29746858 10.1016/j.juro.2018.05.004

[10] S. A. Huang and J. D. Lie , “Phosphodiesterase‐5 (PDE5) Inhibitors in the Management of Erectile Dysfunction,” Pharmaceutics & Therapeutics 38, no. 7 (2013): 407–419. PMC3776492 24049429

[11] R. A. Kloner , J. B. Kostis , T. P. McGraw , C. Qiu , and A. Gupta , “Analysis of Integrated Clinical Safety Data of Tadalafil in Patients Receiving Concomitant Antihypertensive Medications,” Journal of Clinical Hypertension 24, no. 2 (2022): 167–178, 10.1111/jch.14435. 35099113 PMC8845471

